# The impact of active mentorship: results from a survey of faculty in the Department of Medicine at Massachusetts General Hospital

**DOI:** 10.1186/s12909-018-1191-5

**Published:** 2018-05-11

**Authors:** Rochelle P. Walensky, Younji Kim, Yuchiao Chang, Bianca C. Porneala, Mirar N. Bristol, Katrina Armstrong, Eric G. Campbell

**Affiliations:** 1 Medical Practice Evaluation Center, Massachusetts General Hospital, 100 Cambridge Street, 16th floor, Boston, MA 02114 USA; 2 Division of Infectious Diseases, Massachusetts General Hospital, Boston, MA USA; 3 General Internal Medicine, Massachusetts General Hospital, Boston, MA USA; 4 Department of Medicine, Massachusetts General Hospital, Boston, MA USA; 5 Mongan Institute Health Policy Center, Massachusetts General Hospital, Boston, MA USA; 6 Harvard Medical School, Boston, MA USA

**Keywords:** Faculty survey, Mentor, Mentee, Job satisfaction, Academic advancement, Academic rank

## Abstract

**Background:**

To assess mentorship experiences among the faculty of a large academic department of medicine and to examine how those experiences relate to academic advancement and job satisfaction.

**Methods:**

Among faculty members in the Massachusetts General Hospital Department of Medicine, we assessed personal and professional characteristics as well as job satisfaction and examined their relationship with two mentorship dimensions: (1) *currently have a mentor* and (2) *role as a mentor*. We also developed a mentorship quality score and examined the relationship of each mentorship variable to academic advancement and job satisfaction.

**Results:**

553/988 (56.0%) of eligible participants responded. 64.9% reported currently having a mentor, of whom 21.3% provided their mentor a low quality score; 66.6% reported serving as a mentor to others. Faculty with a current mentor had a 3.50-fold increased odds of serving as a mentor to others (OR 3.50, 95% CI 1.84–6.67, *p* < 0.001). Faculty who reported their mentorship as high quality had a decreased likelihood of being stalled in rank (OR 0.28, 95% CI: 0.10–0.78, *p* = 0.02) and an increased likelihood of high job satisfaction (OR 3.91, 95% CI 1.77–8.63, *p* < 0.001) compared with those who reported their mentorship of low quality; further, having a low mentorship score had a similar relationship to job satisfaction as not having a mentor.

**Conclusions:**

A majority of faculty survey respondents had mentorship, though not all of it of high caliber. Because quality mentorship significantly and substantially impacts both academic progress and job satisfaction, efforts devoted to improve the adoption and the quality of mentorship should be prioritized.

**Electronic supplementary material:**

The online version of this article (10.1186/s12909-018-1191-5) contains supplementary material, which is available to authorized users.

## Background

Derived from the root “men” meaning “to think”, *Mentor* was the trusted friend of Odysseus who oversaw Odysseus’ affairs in his absence. Since the times of Greek mythology, mentorship has evolved in academia as the practice of guiding careers through provision of role modeling, resources, connections and advice. When operating optimally, mentoring is mutually beneficial, enhancing knowledge, satisfaction and quality work performance of both parties [1].

Over the last several decades, mentorship has been increasingly recognized as essential to successful academic advancement and job satisfaction [2–4]. Surveys of female medical faculty members in the mid-1980s found that < 20% ever had a mentor during their academic tenure [5]. Since then, several large multi-institutional surveys have demonstrated a modest rise in the frequency of mentorship (30–54%) [6–8].

Given its critical import and lack of universal adoption in academia, we embarked upon a plan toward development of a systematic, empirically-based mentoring culture in the Department of Medicine at the Massachusetts General Hospital (MGH) —a large teaching hospital affiliated with Harvard Medical School (HMS). We began by conducting a mentorship needs assessment among the nearly 1000 full-time faculty in the department. Using a self-administered, electronic survey of faculty, we sought to examine the factors associated with mentorship, with faculty serving in mentorship roles to others, and with mentorship quality. We also examined how these mentorship variables are associated with academic advancement and overall job satisfaction.

## Methods

### Survey instrument and variables

We developed a 12-page survey that took approximately 15 min to complete. Survey items were developed in one of three ways: some were taken from previously administered faculty surveys; some were modified from existing items; and some were developed de novo. Questions were related to the following domains: personal demographics; professional characteristics; job satisfaction; mentoring; and prospects for promotion (as well as other domains not reported here). Personal characteristics measured on the survey included (among others), gender, race, ethnicity, and number of dependent children (< 21 years). The race variable was grouped into two categories—under-represented minority in medicine or non-under-represented minority in medicine (White/Asian). Gender was also collapsed into two categories—male or female. Professional characteristics included academic rank (instructor, assistant professor, associate professor, or professor) and Harvard Medical School (HMS) promotional pathway (Investigator, Clinical Expertise and Innovation, or Teaching and Educational Leadership).

While the MGH Department of Medicine endorses, encourages, and facilitates mentorship, there were no established formal mentorship assignments at the time of this survey. Mentorship questions were thus intended to assess faculty perceptions related to what a mentor traditionally provides, rather than the examination of a conventional program or intervention. In the survey, mentorship was defined as “someone who serves as a career role model and who advises, guides and promotes his or her mentee’s career or training.” Experiences with mentorship were measured through individual questions about whether faculty currently had a mentor or served as a mentor to someone else and through a series of questions asking the quality of their current mentors’ performance. Faculty evaluated the quality of their mentors on a 5-point Likert scale in a total of ten areas, ranging from assistance they received in review of scientific work or grants to provision of opportunities for career advancement to advice about work/family balance (See Additional file 1 for a copy of the survey) [9].

### Survey sample

Email contact information for all faculty in the Department of Medicine at MGH was obtained from the Department of Medicine faculty affairs tracking database which includes data on HMS professorial rank (instructor to professor). Of the faculty identified in these databases, we identified 988 holding full-time faculty appointments in the MGH Department of Medicine and HMS.

### Survey administration and response rate

We received Partners HealthCare Human Subjects Committee Exemption (Protocol Number 2016P000935) to conduct the online survey. The survey was first disseminated via email invitation on June 1, 2016, using the Research Electronic Data Capture (REDCap) secure, web-based application hosted at Massachusetts General Hospital [10]. Each eligible participant was provided a unique link for survey completion; no incentives were offered. Non-respondents were sent survey reminders and their individual links on June 8, 15, and 27; the survey was closed on June 30, 2016. Among the 988 eligible participants, 553 completed the survey yielding an overall response rate of 56.0%. Once data collection was complete, all information linking individual faculty to their survey responses was destroyed, creating a completely anonymous dataset.

### Data analysis

To isolate the impact of active ongoing mentorship, respondents were categorized as currently having a mentor, previously having a mentor (but not currently), or never having a mentor. For those who reported they currently had a mentor, we inquired about mentorship quality (those without a current mentor were excluded). To evaluate quality, the survey inquired “in your experience at MGH, how well has your mentor(s) done in providing…” We then asked faculty to consider areas, among others, including review of scientific work, assistance in grant writing, advice about promotions, advice as a clinician, researcher or teacher, and opportunities for career advancement (see Additional file 1, Section E for complete list of quality questions). Because mentorship quality evaluated a diverse menu of mentorship activities – not all of which were relevant for all promotional pathways – we included scores of those who responded to at least 5 of 10 questions related to their mentor’s performance. We then created a mentorship score by averaging the results from the responded questions. Of a maximum average score of 5, we dichotomized results as low (< 3) and high (≥ 3) scores.

Excluding professors, we combined data from questions related to current academic rank and year of appointment to that rank, to estimate the median duration of time spent in each rank. To examine the relationship of mentorship to academic progress, we created a variable “*stalled in rank” (stalled)* where *stalled* faculty members were those who have spent more than three times the median number of years in their current academic rank (mean and median results were similar for this variable). With this definition, a faculty member was considered stalled if s/he was an instructor for > 9 years, an assistant professor for > 12 years, and an associate professor for > 15 years.

While we inquired about many aspects of job satisfaction, we measured job satisfaction with the single global survey item, “Overall how satisfied are you with your current position at MGH?” Responses from the 5-point Likert scale (very unsatisfied, unsatisfied, neither, satisfied, very satisfied) were collapsed to three categories for analysis: strongly satisfied, somewhat satisfied and not satisfied (inclusive of all other answers).

We considered four outcome variables for analysis: *currently have a mentor, role as a mentor, stalled in rank* and *job satisfaction.* The common predictors for all outcomes were gender, race, professional rank, and academic pathway. Depending on the outcome, we also considered other predictors that might have potential effects on the particular outcome, which included currently have a mentor, having any dependents, and mentorship score. Currently have a mentor and stalled were also evaluated as predictors for overall satisfaction.

Using univariate analysis, we examined the distribution of each survey item. In bivariate analysis, we used Chi-square tests to assess any relationship between the mentorship outcome variables and predictor variables. To test the independent effects of each predictor, we conducted multivariate analyses of the two outcomes using logistic regression models. Missing data were excluded from the analysis using the listwise deletion approach. All statistical tests were two-sided with a significance level of < 0.05. Analyses were performed using SAS version 9.4 (SAS Institute, Cary, NC).

## Results

### Characteristics of the respondents

Of the 553 respondents, 55.7% were male, similar to the male proportion of faculty in the MGH Department of Medicine (57.8%, Table 1). Largely corresponding to the departmental rank distribution, instructors represented 44.6%, assistant professors 30.4%, associate professors 14.4%, and professors 10.6% of all respondents. Most respondents were on the Investigator pathway (45.0%), followed by the Clinical Expertise (37.7%) and the Teaching and Educational Leadership (17.2%) pathways.

**Table 1:** Cohort characteristics

	Respondents (*N* = 553)		MGH DOM^a^(*N* = 988)
	*N*	% ^b^	%^a^
*Demographic characteristics:*			
Gender^a^			
Male	308	55.7	57.8
Female	245	44.3	42.2
Race			
Non-minority^c^	514	94.0	N/A
Minority	33	6.0	N/A
Have any dependents			
No	213	39.2	N/A
Yes	330	60.8	N/A
*Professional characteristics:*			
Rank^a^			
Instructor	245	44.6	50.3
Assistant Professor	167	30.4	26.1
Associate Professor	79	14.4	13.9
Professor	58	10.6	9.7
Pathway			
Investigator	222	45.0	N/A
Non-investigator	271	55.0	N/A
Have a mentor at MGH			
Never	96	20.9	N/A
Currently	298	64.9	N/A
Previously	65	14.2	N/A
Mentorship scored^d^			
Low (< 3)	59	21.3	N/A
High (≥3)	218	78.7	N/A
Serves as a mentor to others			
Yes	325	66.6	N/A
No	163	33.4	N/A
Primary mentor is person to whom I report^d^			
No	94	32.0	N/A
Yes	200	68.0	N/A
Stalled^e^			
Yes	70	15.8	N/A
No	374	84.2	N/A

*MGH* Massachusetts General Hospital, *DOM* Department of Medicine

^a^For the DOM population, gender and rank were from the faculty affairs tracking database; race and promotional pathway were not available from this database. For the survey respondents, gender and rank were from survey responses, supplemented (i.e. missing data) by tracking database

^b^Percentages among respondents to each characteristic question

^c^Non-minority includes Whites and Asians

^d^Mentorship score and whether or not a primary mentor is a direct supervisor were assessed only among faculty who currently have a mentor

^e^The stalled variable excludes faculty with the rank of Professor

### Currently have a mentor at MGH

Excluding those who had a mentor in the past, 74.3% of the respondents reported they currently have a mentor at the MGH. In bivariate analyses, rates of current mentorship did not vary significantly by gender or minority status. However, significantly more instructors (83.1%) reported currently having a mentor compared to assistant (69.0%), associate (63.0%) or full professors (64.1%, *p* = 0.002). Further, compared to faculty on the HMS Investigator promotional pathway, fewer reported currently having a mentor at MGH if they were on the non-investigator pathways (61.7% vs. 89.3% *p* < 0.001, Table 2, Left).

**Table 2:** Predictors of currently having a mentor at MGH^a, b^

	*Bivariate Analysis*		*Multivariate Analysis*		
	Yes, n/N (%)	*p*-value	OR	95% CI	*p*-value
Gender					
Male	152/209 (72.7)	0.45	Ref		
Female	146/192 (76.0)		1.15	0.68–1.93	0.61
Race					
Non-minority	276/372 (74.2)	0.84	Ref		
Minority	19/25 (76.0)		0.99	0.35–2.82	0.99
Rank					
Instructor	157/189 (83.1)	0.002	Ref		
Assistant Professor^c^	87/126 (69.0)		0.40	0.22–0.73	0.002
Associate Professor	29/46 (63.0)		0.30	0.14–0.68	0.004
Professor	25/39 (64.1)		0.17	0.07–0.42	< 0.001
Pathway					
Investigator	158/177 (89.3)	< 0.001	Ref		
Non-investigator	127/206 (61.7)		0.15	0.08–0.27	< 0.001

^a^Outcome variable was assessed by asking the following question: “Do you currently have at least one person at Massachusetts General Hospital who you consider to be mentor?”

^b^This analysis excluded those who noted they had a mentor in the past (but not currently)

^c^When Assistant Professors were used as the referent group, there was no statistically significant difference between Associate Professors and Assistant Professors (OR 0.75, 95% CI 0.34–1.68) but there was a trend toward Professors less frequently having a current mentor than Assistant Professors (OR 0.43 95% CI 0.17–1.04)

In the multivariate model that included gender, race, rank and academic pathway, assistant professors, associate professors, and professors had over a 2-fold decreased odds of currently having a mentor compared to instructors. When assistant professors were used as the referent group, associate professors and professors did not demonstrate a significant difference in the odds of currently having a mentor at MGH. Similar results were demonstrated for those on the non-investigator pathways (OR 0.15, 95% CI: 0.08–0.27, *p* < 0.001) in that they had over a 6-fold decreased odds of currently having a mentor, compared to those on the investigator pathway (Table 2, Right).

### Role as a mentor

Overall, 325 (66.6%) of faculty respondents reported serving as a mentor to someone else at the MGH. In bivariate analysis, the frequency of serving as a mentor did not vary significantly by race, but did by many other demographics variables examined including: gender (male 74.5%, female 56.2%, *p* < 0.001); academic rank (ranging from instructor 50.0% to professor 94.6%, *p* < 0.001); promotional pathway (investigator 77.4%, non-investigator 59.5%, *p* < 0.001) and whether the faculty currently had a mentor him/herself (never 50.0%, yes 67.7% and previously 75.0%, *p* = 0.001).

In multivariate analyses, females remained less likely to serve as a mentor (0.57, 95% CI 0.35–0.91, *p* = 0.02). Compared to instructors, assistant professors (3.40, 95% CI: 2.00–5.79, *p* < 0.001), associate professors (11.5, 95% CI: 4.35–30.5, *p* < 0.001), and professors (19.8, 95% CI: 5.51–71.4, *p* < 0.001) were all more likely to serve as a mentor, in a dose-response fashion (Table 3, Right). Further, those who reported having a mentor themselves had a 3.5-times increased odds of mentoring others (OR 3.50, 95% CI: 1.84–6.67, *p* < 0.001). Faculty on non-investigator promotional pathways also tended to have less frequently served as a mentor, though this bordered on statistical significance. However, serving as a mentor was not significantly associated with a faculty member being stalled in rank (OR 0.93, 95% CI: 0.47–1.84, *p* = 0.83), after controlling for other factors.

**Table 3:** Predictors of having a role as a mentor at MGH^a^

	*Bivariate Analysis*		*Multivariate Analysis*		
	Yes, n/N (%)	*p*-value	OR	95% CI	*p*-value
Gender					
Male	207/278 (74.5)	< 0.001	Ref		
Female	118/210 (56.2)		0.57	0.35–0.91	0.02
Race					
Non-minority	303/454 (66.7)	0.53	Ref		
Minority	19/31 (61.3)		1.62	0.67–3.91	0.28
Rank					
Instructor	107/214 (50.0)	< 0.001	Ref		
Assistant Professor	104/150 (69.3)		3.40	2.00–5.79	< 0.001
Associate Professor	60/67 (89.6)		11.5	4.35–30.5	< 0.001
Professor	53/56 (94.6)		19.8	5.51–71.4	< 0.001
Pathway					
Investigator	161/208 (77.4)	< 0.001	Ref		
Non-investigator	153/257 (59.5)		0.63	0.38–1.04	0.07
Have a mentor at MGH					
Never	49/98 (50.0)	0.001	Ref		
Currently	197/291 (67.7)		3.50	1.84–6.67	< 0.001
Previously	48/64 (75.0)		2.88	1.19–6.95	0.02
Stalled^b^					
No	228/351 (65.0)	0.09	Ref		
Yes	35/65 (53.8)		0.93	0.47–1.84	0.83

^a^Outcome variable was assessed by asking the following question “How many people do you currently mentor?” If faculty had at least one mentee, then they were considered having a role as a mentor

^b^Excluding those who had a mentor in the past (not currently) and those of professor rank

### Mentorship as it relates to stalled in rank

Among respondents, we found 15.8% of faculty were stalled in rank (Table 1). In bivariate analysis, compared to faculty who had mentors with a high mentorship score (5.6%), faculty without mentors (26.6%), those who only previously had a mentor (28.0%), or those whose mentors had a low score (14.8%) were significantly more likely to be stalled (*p* < 0.001, see Table 4, left, for other significant associations). In multivariate analysis, faculty who rated their mentor with a high score had over 3.5-fold less likely odds of being stalled (OR 0.28, 95% CI: 0.10–0.78, *p* = 0.02); and faculty who never had a mentor were even more likely to be stalled compared to those providing a low mentorship score (3.38, 95% CI 1.13–10.2, *p* = 0.03, Table 4, right).

**Table 4:** Predictors for stalled in rank^a^

	*Bivariate Analysis*		*Multivariate Analysis*		
	Yes, n/N (%)	*p*-value	OR	95% CI	*p*-value
Gender					
Male	41/233 (17.6)	0.27	Ref		
Female	29/211 (13.7)		0.77	0.39–1.53	0.46
Race					
Non-minority	69/409 (16.9)	0.04	Ref		
Minority	1/33 (3.0)		0.17	0.02–1.36	0.09
Rank ^b^					
Instructor	43/225 (19.1)	0.13	Ref		
Assistant Professor	17/148 (11.5)		0.52	0.24–1.11	0.09
Associate Professor	10/71 (14.1)		0.19	0.06–0.66	0.009
Pathway					
Investigator	14/182 (7.7)	< 0.001	Ref		
Non-investigator	49/238 (20.6)		1.42	0.62–3.25	0.40
Have any dependents					
No	36/156 (23.1)	0.002	Ref		
Yes	33/282 (11.7)		0.57	0.29–1.13	0.11
Mentorship Score					
Low (< 3)	8/54 (14.8)	< 0.001	Ref		
High (≥3)	11/195 (5.6)		0.28	0.10–0.78	0.02
Previously had a mentor at MGH	14/50 (28.0)		1.81	0.64–5.10	0.26
Never had a mentor at MGH	21/79 (26.6)		3.38	1.13–10.2	0.03

^a^Outcome was measured by subtracting the year when current rank was assigned from current year 2016. A faculty was considered stalled if one has been in the current rank for more than three times the median time in a given range; that is, > 9 years for an instructor, > 12 years for an assistant professor, and > 15 years for an associate professor

^b^Professors were excluded in the analysis

### Mentorship at it relates to job satisfaction

Of the respondents, almost a third (31.5%) of faculty reported that they are ‘strongly satisfied’ with their current position. In bivariate analysis, responses to the satisfaction question were significantly associated with currently having a mentor and the mentorship quality score (Table 5, left, for other significant associations). In multivariate analysis, those with high mentorship scores had nearly a 4-fold odds of reporting strong job satisfaction (OR 3.91, 95% CI 1.77–8.63, *p* < 0.001, Table 5, right).

**Table 5:** Job satisfaction

	*Bivariate Analysis*				*Multivariate Analysis* ^b^		
	Not satisfied,^a^ N (%)	Somewhat satisfied, N (%)	Strongly satisfied, N (%)	*p*-value	OR	95% CI	*p*-value
Gender							
Male	62 (21.8)	117 (41.2)	105 (37.0)	0.009	Ref		
Female	63 (29.0)	101 (46.5)	53 (24.4)		0.48	0.30–0.77	0.002
Race							
Non-minority	115 (24.6)	204 (43.6)	149 (31.8)	0.96	Ref		
Minority	8 (26.7)	13 (43.3)	9 (30.0)		1.08	0.44–2.63	0.86
Rank							
Instructor	63 (28.3)	93 (41.7)	67 (30.0)	0.002	Ref		
Assistant Professor	45 (29.6)	69 (45.4)	38 (25.0)		0.88	0.52–1.50	0.63
Associate Professor	10 (14.5)	35 (50.7)	24 (34.8)		1.30	0.65–2.60	0.46
Professor	7 (12.5)	20 (35.7)	29 (51.8)		2.78	1.35–5.74	0.006
Pathway							
Investigator	48 (22.5)	89 (41.8)	76 (35.7)	0.37	Ref		
Non-investigator	64 (24.6)	119 (45.8)	77 (29.6)		1.00	0.63–1.58	0.99
Mentorship Score							
Low (< 3)	27 (46.6)	21 (36.2)	10 (17.2)	< 0.001	Ref		
High (≥3)	37 (17.1)	92 (42.4)	88 (40.6)		3.91	1.77–8.63	< 0.001
Previously had a mentor at MGH	17 (27.0)	25 (39.7)	21 (33.3)		1.88	0.73–4.85	0.19
Never had a mentor at MGH	34 (33.0)	50 (48.5)	19 (18.4)		0.99	0.39–2.50	0.98

^a^Included all responses of very unsatisfied, unsatisfied, or neither satisfied nor unsatisfied

^b^Outcome of multivariate analysis: strongly satisfied

## Discussion

Among faculty in the Department of Medicine at the MGH, we found substantial variation in mentorship experiences. Mentorship deficiencies are most notable among faculty who are not on the research “Investigator” promotion pathway. That is, faculty whose careers are focused on “Clinical Education and Innovation” or “Teaching and Educational Leadership” are among the faculty most likely not to have mentorship. Given that the National Institutes of Health has an embedded component of mentorship through the K-series awards, these results are not surprising; mentors are, by definition, a required peer-reviewed component of federal funding, funding which is fundamental toward investigator independence [11]. However, in these other important academic pathways, such national mentorship standards are less institutionalized and fostered; these non-investigator pathways might similarly benefit from clearer roles with respect to mentorship, including academic promotion related to the success of their mentees.

We also find that faculty who had mentors themselves were over 3-fold more likely to provide mentorship to others. As faculty on either the clinical or teaching academic pathways were less likely to have mentors themselves, trends suggested that they were subsequently and independently less likely to serve as one. These results imply the need for investments in mentoring activities targeted to faculty with these alternative but critical academic missions of patient care and medical education to break this potentially perpetuating cycle.

When we examine mentoring quality, we find that 21.3% of faculty report relatively low mentor quality. Research suggests that quality mentorship in academic medicine is essential to faculty productivity, job satisfaction and retention [4, 7, 8]. Our results are consistent with, and further enhance, these findings. We demonstrate that academic advancement – using a newly-defined *stalled in rank* surrogate definition – is significantly and inversely associated with scores of excellence in mentorship. Faculty with high-quality mentors are significantly less likely to be stalled in rank. Additionally, we find that faculty with low-quality mentors fare similarly to those without a mentor at all. In a similar vein, faculty who report high-quality mentors are nearly 4-fold more likely to also strongly endorse high job satisfaction. Faculty who endorse poor-quality mentors also report poor job satisfaction, and those who never had a mentor fare even worse on this satisfaction outcome.

While our study focused on having any mentor, there is a growing literature suggesting that a single mentor is, in fact, not enough. Many such “dyad” mentoring programs – where faculty are paired with individual protégés – are met with only variable success, and likely reporting bias [12–14]. More frequently touted now are the preference for mentorship teams, where mentees are able to draw from a menu of more senior faculty who offer diverse opinions, backgrounds and areas of expertise and who collectively offer a wide representation of potentially tap-able networks [11, 15]. The paucity of currently available mentors, the need for several mentors for each individual, the expansion of the numbers of faculty needing mentorship, and the productivity pressures on today’s faculty – both junior and senior – can create the current mentorship supply and demand problems that may contribute to our survey results [2].

While the concept of “mentorship malpractice” – where a mentoring relationship is counterproductive and even harmful to a mentee – is well-documented [16], less well-described is “mentorship benign neglect.” Our study finds that faculty who report having no current mentor (36%) fare similarly to those with poor quality mentors on the important outcome of job satisfaction. These findings emphatically highlight that simply “having a mentor” is not the only operative question. The quality of mentorship – with regard to issues of faculty support, networking and promotional advancement – must be addressed to impact this outcome. Thus, to fix the composite issue, faculty need to not only engage in mentoring but they need to be trained to do it well. Research has demonstrated that faculty who participate in such “train the mentor” programs become both more engaged and more successful mentors [12, 17–20].

Despite the frequently reported request among mentees for their mentors to be of similar gender and/or race [11], when adjusted for other important factors, we were unable to detect any significant association related to issues of current mentorship, gender and race. We consider these results promising, given the relative paucity of senior women and minority faculty at the MGH and similar academic institutions [6]. Quality mentorship can cross both gender and racial lines and, in fact, both parties would likely benefit from such lack of concordance. We were unfortunately unable to examine other issues of diversity – such as sexual orientation and disability – due to small sample sizes.

In a question about job satisfaction, about one-third of faculty reported they were “very satisfied” and slightly more than half reported they were at least “somewhat satisfied”. While these statistics are disappointingly low, they are similar to recent national physician survey data demonstrating overall career satisfaction of 45–65%, depending on medical specialty [21]. In our study, satisfaction was associated with quality mentorship, though it likely has other key contributing factors as well, including work hours, administrative burden, and salary. In one Veterans Affairs study, involvement in research was associated with increased physician satisfaction [22]. To the extent that researchers in the Department of Medicine benefit from higher quality mentors, our results are potentially consistent with this study.

Our findings should be interpreted within the context of several limitations. First, results are subject to the inherent reporting biases that often occur in survey studies. To our best ability to assess, we appear reasonably balanced in response rate by gender and academic rank; however, we cannot evaluate important potential response biases associated with promotional pathways and mentorship experiences. Second, our results may not be generalizable beyond the Department of Medicine at the MGH. However, we hope these results will motivate other institutions to consider their own mentorship evaluative studies and further propagate national attention to these needs broadly in academic medicine. Third, we acknowledge that results of the survey – especially with regard to career advancement and job satisfaction – may be influenced and confounded by mentorship that occurs from individuals outside of MGH. Finally, we acknowledge that our assessment of *stalled in rank* as a surrogate for academic productivity is limited: MGH does not have the typical “up or out” tenure process, and HMS has a well-recognized protracted process of promotional advancement. Further, those on the Clinical Expertise pathway who are more motivated by excellent, devoted patient care than by promotion may not select to spend their energy on a time-intensive promotional process.

We are among the first to empirically “open the box” on mentoring activities at the MGH, examining the difficult question within our own department, “how are we doing?” We find that two-thirds of our faculty – especially those involved in scientific investigation – are supported by thriving mentor relationships, and these faculty are met with similar successful academic productivity and job satisfaction. The remaining faculty represent potential opportunities lost that must be redeemed.

## Conclusions

Having identified areas needing attention, we intend to take the critical next steps toward improvement and change. Central to these steps will be the establishment of mentorship development activities, both to expand the pool of available mentors and to improve the effectiveness of ongoing mentorship activities. Identification of current mentorship successes in pathways other than investigation will be a key toward understanding and promoting these relationships toward both emulation and expansion.

## Additional file

Additional file 1: Copy of survey directed to Massachusetts General Hospital Department of Medicine Faculty. Survey domains include: personal demographics, professional characteristics, diversity, overall satisfaction, mentoring, prospects for promotion, relationship with supervisor/chief, and comments and additional demographics. (PDF 174 kb)

## Abbreviations

HMS: Harvard Medical School
MGH: Massachusetts General Hospital
REDCap: Research Electronic Data Capture
