# Supplementary material for: The impact of active mentorship: results from a survey of faculty in the Department of Medicine at Massachusetts General Hospital
Source: BMC Med Educ. 2018 May 11;18:108. doi: 10.1186/s12909-018-1191-5 (PMC5948924; doi:10.1186/s12909-018-1191-5)
Supplement: Supplementary file 1 — Copy of survey directed to Massachusetts General Hospital Department of Medicine Faculty. Survey domains include: personal demographics, professional characteristics, diversity, overall satisfaction, mentoring, prospects for promotion, relationship with supervisor/chief, and comments and additional demographics. (PDF 174 kb) [file 12909_2018_1191_MOESM1_ESM.pdf]

MASSACHUSETTS GENERAL HOSPITAL

Resize font:

About this survey:

This survey is directed to faculty in the **MGH Department of Medicine** and asks about issues related to diversity, mentorship and satisfaction. We intend to use the survey results to prioritize areas of action across the department and as a baseline to follow improvements related to these activities over time.

Survey responses will be kept confidential; no data will be released that allows for identification of any single individual. Presentation of the data will be limited to respondent groups of five or more. We value your candid input so that we can direct our activities accordingly. If, however, you deem some questions too sensitive, please feel free to skip them and to selectively respond to those with which you are comfortable.

We appreciate your participation toward the development of the department, as a whole.

Submit

REDCap Software - Version 6.10.11 - © 2016 Vanderbilt University

Section A: Personal Demographics

1. Do you hold a faculty appointment at Harvard Medical School?
   - ◯ Yes
   - ◯ No

2. Is your primary appointment in the Department of Medicine at MGH?
   - ◯ Yes
   - ◯ No

---

3. Please indicate your marital status.
   - ◯ Single
   - ◯ Living with a partner
   - ◯ Married
   - ◯ Separated/Divorced
   - ◯ Widowed
   - ◯ Other

Please specify: ______________________________

4. How many children under the age of 21 currently reside in your home? (Please enter "0" if none.)

____
(Please enter zero if none.)

5. Please indicate what degrees you hold. Please check all that apply.
   - ☐ MD
   - ☐ PhD
   - ☐ Other doctorate
   - ☐ Other

Please specify: ______________________________

6. Please indicate your ethnicity.
   - ◯ Hispanic
   - ◯ Non-Hispanic

7. Please indicate your race.
   - ◯ American Indian or Alaska Native
   - ◯ Asian
   - ◯ Black or African American
   - ◯ Native Hawaiian or Other Pacific Islander
   - ◯ White
   - ◯ Other

Please specify: ______________________________

8. Please indicate your gender.
   - ◯ Male
   - ◯ Female
   - ◯ Transgender Male
   - ◯ Transgender Female
   - ◯ Gender-expansive
   - ◯ Other

Please specify: ______________________________

Section B: Professional Characteristics

1. In what division of the Department of Medicine at MGH are you appointed?

- ◯ Cardiology
- ◯ Endocrine
- ◯ Gastroenterology
- ◯ General Internal Medicine
- ◯ Hematology & Oncology
- ◯ Infectious Diseases
- ◯ Nephrology
- ◯ Palliative Care
- ◯ Pulmonary & Critical Care Medicine
- ◯ Rheumatology, Allergy and Immunology
- ◯ Other

Please specify: ______________________________

2. Do you hold a faculty appointment in any of the following research units, centers, or institutes? Please check all that apply.

- ☐ Analytic and Translational Genetics Unit
- ☐ Biostatistics Center
- ☐ Broad Institute
- ☐ Center for Assessment Technology and Continuous Health
- ☐ Center for Immunology and Inflammatory Diseases
- ☐ Clinical and Translational Epidemiology Unit
- ☐ Disparities Research Unit
- ☐ Laboratory of Computer Science
- ☐ Medical Practice Evaluation Center
- ☐ Mongan Institute Health Policy Center
- ☐ Ragon Institute
- ☐ Vaccine and Immunotherapy Center
- ☐ None of above

If other, please specify: ______________________________

3. In what year were you first appointed to the faculty at Harvard Medical School?

____
(YYYY)

3a. At what rank were you first appointed to the faculty at Harvard Medical School?

- ◯ Instructor
- ◯ Assistant Professor
- ◯ Associate Professor
- ◯ Professor

4. Please indicate your current academic rank at Harvard Medical School.

- ◯ Instructor
- ◯ Assistant Professor
- ◯ Associate Professor
- ◯ Professor

4a. In what year were you appointed to your current rank at Harvard Medical School?

____
(YYYY)

5. Please indicate which HMS promotion track you are on.

- ◯ Clinical Expertise and Innovation
- ◯ Investigator
- ◯ Teaching and Educational Leadership

6. Would you like to change your HMS promotion track in the future?

- ◯ Yes
- ◯ No

6a. If yes, which HMS promotion track would you like to be on?

- ◯ Clinical Expertise and Innovation
- ◯ Investigator
- ◯ Teaching and Educational Leadership

Distribution of professional time:

7. During a typical work week, about how many hours in total do you spend on professional activities?

______________________ (Number of Hours)

Note: Professional activities include patient care, research, teaching, consulting, professional travel, writing, professional reading, and administration. Please exclude time on call when not actually working.

In a typical work week, about what percentage (%) of the total hours entered above do you spend in each of the following professional activities? (Please enter "0" if you don't engage in an activity.)

7a. In direct patient care services:

______________________ (%)

Description: Include all time you spend directly related to patient care, patient record keeping, patient related office work, travel time connected with seeing patients. Please exclude time on call when not actually working.

7b. In all teaching activities:

______________________ (%)

Description: Include your instruction of students, residents, interns and fellows, in all clinical, laboratory and classroom settings.

7c. In all research activities:

______________________ (%)

Description: Include all time spent writing proposals, papers, and reports, time spent planning and executing projects, and any laboratory time.

7d. In administrative activities:

______________________ (%)

Description: Include clinical and academic administration, faculty and departmental meetings, and committees.

7e. In professional activities outside your institution:

______________________ (%)

Description: Include consulting to outside groups, companies and agencies, professional society activities, lecturing to outside groups, employment in professionally related companies, and travel associated with the above.

Total Hours

______________________ (%)

Section C: Diversity

1. To what extent do you agree or disagree that the Department of Medicine at MGH encourages and embraces diversity?

- ◯ Strongly disagree
- ◯ Somewhat disagree
- ◯ Neither agree nor disagree
- ◯ Somewhat agree
- ◯ Strongly agree

2. To what extent do you agree or disagree that the Department of Medicine at MGH treats you with dignity and respect?

- ◯ Strongly disagree
- ◯ Somewhat disagree
- ◯ Neither agree nor disagree
- ◯ Somewhat agree
- ◯ Strongly agree

Please rate the extent to which you believe your work at MGH has been hampered by discrimination related to...

	To a great extent	To some extent	Very little	Not at all
3. Gender	◯	◯	◯	◯
4. Race, ethnicity, or religion	◯	◯	◯	◯
5. Sexual orientation	◯	◯	◯	◯
6. Disability	◯	◯	◯	◯

Please rate the extent to which you believe your career advancement at MGH has been hampered by discrimination related to...

	To a great extent	To some extent	Very little	Not at all
7. Gender	◯	◯	◯	◯
8. Race, ethnicity, or religion	◯	◯	◯	◯
9. Sexual orientation	◯	◯	◯	◯
10. Disability	◯	◯	◯	◯

Section D: Overall Satisfaction

Please rate the extent to which you agree or disagree with each of the following statements:

	Strongly disagree	Somewhat disagree	Neither agree nor disagree	Somewhat agree	Strongly agree
1. On average, I look forward to going to work.	○	○	○	○	○
2. There must be better places to work.	○	○	○	○	○
3. My job means more to me than just money.	○	○	○	○	○
4. I am satisfied with the work I do.	○	○	○	○	○
5. I feel as if I belong here.	○	○	○	○	○
6. The work I do here is intellectually fulfilling.	○	○	○	○	○

Please rate the extent to which you agree or disagree with each of the following statements about the leadership in your division:

The leadership in my division...

	Strongly disagree	Somewhat disagree	Neither agree nor disagree	Somewhat agree	Strongly agree
7. cares about me as a person.	○	○	○	○	○
8. values what I do.	○	○	○	○	○
9. takes me for granted on a regular basis.	○	○	○	○	○
10. provides me with sufficient opportunities for professional growth.	○	○	○	○	○
11. provides the necessary resources so I can take advantage of opportunities for professional growth.	○	○	○	○	○
12. has a transparent process in determining compensation.	○	○	○	○	○
13. has a fair process in determining compensation.	○	○	○	○	○

14. compensates me fairly.	◯	◯	◯	◯	◯

---

15. Overall how satisfied are you with your current position at MGH?

◯ Very satisfied
◯ Satisfied
◯ Neither satisfied nor unsatisfied
◯ Unsatisfied
◯ Very unsatisfied

Section E: Mentoring

1. Do you currently have at least one person at MGH who you consider to be mentor?	◯ Yes<br>◯ No
1a. Have you ever had at least one person at MGH who you consider to be your primary mentor?	◯ Yes<br>◯ No

In your experience at MGH, how well have your mentor(s) done in providing the following:

	Poor	Fair	Good	Very good	Excellent	N/A
2a. Review of your scientific work	◯	◯	◯	◯	◯	◯
2b. Assistance to you in writing grants	◯	◯	◯	◯	◯	◯
2c. Advice about your academic promotion	◯	◯	◯	◯	◯	◯
2d. Advice to you as a clinician	◯	◯	◯	◯	◯	◯
2e. Advice to you as a researcher	◯	◯	◯	◯	◯	◯
2f. Advice to you as a teacher	◯	◯	◯	◯	◯	◯
2g. Advice to you about work/family balance	◯	◯	◯	◯	◯	◯
2h. Opportunities for career advancement	◯	◯	◯	◯	◯	◯
2i. Introductions to individuals who could influence your professional advancement	◯	◯	◯	◯	◯	◯
2j. Advocacy for you with department leadership	◯	◯	◯	◯	◯	◯

Thinking of your primary mentor at MGH, is s/he:

	Yes	No
3a. the person to whom you report?	◯	◯
3b. the same gender as you?	◯	◯
3c. the same ethnicity as you?	◯	◯
3d. the same race as you?	◯	◯

Gender and race/ethnicity in the mentoring dynamic:

4. How important is it that your mentor be the same gender as you?
- ◯ Not at all important
- ◯ Not very important
- ◯ Somewhat important
- ◯ Very important
- ◯ Extremely important

5. How important is it that your mentor be the same ethnicity as you?
- ◯ Not at all important
- ◯ Not very important
- ◯ Somewhat important
- ◯ Very important
- ◯ Extremely important

6. How important is it that your mentor be the same race as you?
- ◯ Not at all important
- ◯ Not very important
- ◯ Somewhat important
- ◯ Very important
- ◯ Extremely important

While at MGH, how often have you felt disadvantaged due to issues of:

	Never	Rarely	Sometimes	Frequently	Always
7a. Ownership of intellectual property	◯	◯	◯	◯	◯
7b. Authorship disputes	◯	◯	◯	◯	◯
7c. Questionable scientific integrity of colleagues	◯	◯	◯	◯	◯

Your role as a mentor:

Definition: a mentor is someone who serves as a career role model and who advises, guides, and
promotes his or her mentee's career or training.

8. How many people do you currently mentor? (Please enter "0" if none.)

____
(Number of Mentee(s))

9. In a typical work week, how many hours do you spend in mentorship activities?

Description: Include all time spent advising others on issues related to their professional / career development and academic activities, including reviewing of their talks, research proposals, and manuscripts.

____
(Hours)

Collegiality and Collaboration:

Please rate the extent to which you agree or disagree with each of the following statements:

	Strongly disagree	Somewhat disagree	Neither agree nor disagree	Somewhat agree	Strongly agree
10a. I work in a social and supportive environment.	◯	◯	◯	◯	◯
10b. I socially interact with colleagues after work hours.	◯	◯	◯	◯	◯
10c. I feel comfortable raising personal and/or family responsibilities when scheduling work-related obligations.	◯	◯	◯	◯	◯

Section F: Prospects for Promotion

1. Overall how likely is it that you will be promoted at HMS in the next two years?

Note: Please skip this question if you are already a professor at HMS.

- ◯ Very unlikely
- ◯ Unlikely
- ◯ Neutral
- ◯ Likely
- ◯ Very likely

2. Overall, how do you rate the speed of your academic progress relative to your peers at MGH?

- ◯ Very slow
- ◯ Slow
- ◯ Average
- ◯ Fast
- ◯ Very fast

3. Overall how likely is it that you will be employed at MGH two years from now?

- ◯ Very unlikely
- ◯ Unlikely
- ◯ Neutral
- ◯ Likely
- ◯ Very likely

Section G: Relationship with Supervisor/Chief

Note: Think of "this person" as someone who is primarily responsible for your promotion and compensation when answering the following questions.

1. Do you have an annual career conference or review with this person?
   - ◯ Yes
   - ◯ No

1a. If no, do you have an annual career conference or review with another person?
   - ◯ Yes
   - ◯ No

2. In an average year, how many times do you meet with this person for mentorship?
   ____

3. In general, how difficult is it to schedule a meeting with this person in a timely manner for mentorship?
   - ◯ Not at all difficult
   - ◯ Not very difficult
   - ◯ Somewhat difficult
   - ◯ Very difficult
   - ◯ Extremely difficult

4. In general, how helpful is this person in making decisions regarding the next steps in your career?
   - ◯ Not at all helpful
   - ◯ Not very helpful
   - ◯ Somewhat helpful
   - ◯ Very helpful
   - ◯ Extremely helpful

5. Overall, what influence has this person had on guiding your career?
   - ◯ Large negative
   - ◯ Somewhat negative
   - ◯ No influence
   - ◯ Somewhat positive
   - ◯ Large positive

Section H: Comments & Additional Demographics

Please use the text box below if you would like us to know anything else about your mentoring experience within the Department of Medicine or MGH.

COMMENT: ______________________________

The following are OPTIONAL demographic questions.

If you are willing, please complete and click "Submit" at any time and/or skip any of the following questions.

1. Do you consider yourself to be:
   - ◯ Heterosexual
   - ◯ Gay or Lesbian
   - ◯ Bisexual
   - ◯ Other

Please specify: ______________________________

2. Do you have serious difficulties with mobility, vision, hearing, or mental or emotional health?
   ◯ Yes ◯ No

3. Are there any accommodations that would make it easier for you to do your job?
   - ◯ Yes
   - ◯ No

Please specify: ______________________________

3a. Do you currently have access to the accommodation?
   - ◯ No
   - ◯ Yes
   - ◯ Access to some but not all of the accommodations I need
